# Ageing and cohort trajectories in mental ill-health: An exploration using multilevel models

**DOI:** 10.1371/journal.pone.0235594

**Published:** 2020-07-09

**Authors:** Lucy Prior, Kelvyn Jones, David Manley

**Affiliations:** 1 School of Geographical Sciences, University of Bristol, Bristol, United Kingdom; 2 Department of Urbanism, Delft University of Technology, Delft, The Netherlands; Erasmus MC Desiderius School, NETHERLANDS

## Abstract

Analyses of health over time must consider the potential impacts of ageing as well as any effects relating to cohort differences. The British Household Panel Survey (BHPS) and Understanding Society longitudinal studies are employed to assess trends in mental ill-health over a 26-year period. This analysis uses cross-classified multilevel models in an exploratory, non-parametric approach to evaluate age and cohort effects net of each other. Mental ill-health evidences an initial worsening trend as people age which then reverses and exhibits improvement in late-middle-age, before declining again in the latter stages of life. There were less defined cohort trends. The modelling technique also reveals the relative importance of the temporal contexts in relation to inter- and intra-individual effects on mental ill-health, demonstrating that the ageing and cohort dimensions explain little variation compared to these more dominant within and between influences. Ultimately, we suggest that researchers would benefit from wider use of this exploratory modelling strategy when evaluating underlying health trends and more research is now needed to explore potential explanations of these baseline trajectories.

## Introduction

The importance of how health changes as people progress through different life stages has long been recognised, across an array of demographic, health and epidemiological fields [1–3]. Examining trajectories of different health dimensions provides insight into later outcomes and, through highlighting divergent patterns, informs our understanding of health disparities between different groups. Assessment of temporal trends can serve as a baseline to the later analysis of the factors which explain patterns of health, following the lifecourse epidemiological tradition of research [1, 4].

### Age

When considering health and the lifecourse isolating the change in health due to ageing processes is often the central aim. For instance, there is a substantial health and ageing literature, concerned with the prospect of healthy ageing and with how different dimensions of health are expected to change for individuals as they progress through the latter stages of life [5–7]. This subject is of particular importance across Western societies that have experienced demographic change, with an increasing shift towards an ageing population [3].

There is an established discourse on the temporal development of mental health and wellbeing over the lifecourse. Studies have suggested wellbeing may follow a u-shaped relationship with age, where younger and older persons show higher wellbeing compared with mid-life [8–10]. Moreover, Blanchflower and Oswald [11] supported a u-shape of mental distress, as proxied by antidepressant use, with the peak of use in middle age. A u-shape relationship with age may also be relevant to the development of mental health over time, depending on the degree to which psychological wellbeing is represented in such measures. However, conflicting evidence has also been presented. For instance, Thomas et al. [12] suggested a linear improvement model of a mental health composite of positive and negative attributes, in a study based on participants from San Diego, California. Dimensions of mental health which incorporate negative outcomes, such as anxiety and depressive symptoms, could be expected to worsen as people age, concurrently with declining physical health. For example, Fiske et al. [13] demonstrated higher depressive symptoms in older individuals in a cross-sectional examination, with a longitudinal analysis continuing to reveal worsening over time for those aged 60 and older. However, they also showed support for a u-shape relationship with age as a middle-aged group did not evidence such a decline over their 9-year follow-up.

Therefore, whilst there is a long history of research which informs on potential patterns of mental health development with age, there continues to be discussion. For instance, some have questioned whether apparent u-shape relationships of mental wellbeing and age are in fact an artefact of inappropriate control variables, such as marital status, which can itself be influenced by wellbeing outcomes [14] rather than genuine age trends. Additionally, the continued dominance of cross-sectional studies complicates the issue; the question remains whether true age effects are being presented or whether cohort and other temporal influences are responsible [15, 16].

### Cohort

Cohort effects are a second temporal influence of substantive interest in studies of health over time. Cohort effects concern impacts on health which arise through the shared characteristics or experiences of those born contemporaneously. Cohort effects may reflect changes in environmental or living conditions, societal change or demographic shifts in cohort populations. For instance, individuals born or growing up during economic recessions, or other periods of socioeconomic or resource uncertainty, may suffer long-term consequences in their adult health. Analysing cohort trends helps ground health trajectories in the social and cultural context in which individuals are embedded, following lifecourse developmental theory [4]. Identifying generational differences may also inform on how the health of different groups progresses over time, helping to understand and act on disparities in health. Recent reports, (see [17]), have sparked renewed discussion on the health of younger generations, inviting questions on cohort effects. The report suggests the current generation of young persons (aged 12–24) are likely to experience negative health consequences in their later lives due to a series of social difficulties they face today. Moreover, a potential burden of mental health issues for young people is also being increasingly evidenced [18], stimulating examination of age and cohort effects.

Following cultural shifts which have been hypothesised to impact on a potential burden of mental health issues for younger generations, researchers have focused specifically on the presence of cohort effects in depression and other mental illness outcomes [19]. For example, an increasingly individualistic society has been postulated to play a role in the rising incidence of depressive symptoms among those frequently referred to as ‘millennials’ [19]. Others have characterised trends of growing depression prevalence as a ‘disease of modernity’ [20]. Twenge [19] used repeated time-points of US survey data on adolescents and young adults, and showed that later cohorts (assessed during the 2000s to 2010s) reported depressive symptoms to a higher degree than their earlier cohort counterparts (evaluated 1980s to 1990s). Using three cross-sectional surveys in 1993, 2000 and 2007, Spiers et al. [21] provided evidence for a male cohort effect in common mental disorders in England, with an increase in prevalence between a cohort born in 1943–1949 and one born in 1950–1956. Evaluation of potential cohort trends using longitudinal data which provides a wide range of cohorts and ages would be a useful addition to the literature.

### Inter-individual and intra-individual

Two other important elements to consider when evaluating health over time are intra- and inter-individual effects. Intra-individual effects concern dependency or changes within individuals over time, whilst inter-individual effects refer to differences between persons. Ageing processes can influence aspects of variation both within and between individuals, whilst cohort effects are an aspect that may only feed into inter-individual differences–a person’s cohort is fixed and cannot change. However, there are a plethora of other factors that will feed into variations of mental ill-health within and between individuals. For example, personal factors such as attitudes and behaviours may impact on mental well-being and illness, as well as life events, status, and other contextual dimensions [22].

The following analysis is not aimed at understanding the action of such predictors, instead it aims to expose a method which can offer an initial, direct assessment of the relative importance of the age and cohort temporal dimensions in relation to total inter- and intra-individual variation. As such, we advocate an exploratory, non-parametric approach to reveal underlying temporal health dynamics and systematic age and cohort variations. We demonstrate this technique through an evaluation of temporal trends in mental ill-health. Mental ill-health is considered in this study to be an underlying continuum representing the degree to which an individual’s psychological, emotional, cognitive and social abilities are impacted upon and is operationalised using a composite score indicative of common mental disorders. In so doing this study aims to progress understanding in health demography of trajectories of this important health dimension in a nationally representative sample from Great Britain.

### Data

To evaluate age and cohort trends in health over time, this study uses the British Household Panel Survey (BHPS) and the UK Household Longitudinal Study (UKHLS) [23–25]. These large-scale household panel surveys provide information on a sample of individuals in the population at all ages as well as on a range of different cohorts over time. Data collection and consent was approved by the University of Essex Ethics Committee. Consent for health linkages was approved at UKHLS Wave 1 by the National Research Ethics Service (NRES) Oxfordshire REC A (08/H0604/124), and at BHPS Wave 18 by the (NRES) Royal Free Hospital and Medical School (08/H0720/60) and at Wave 4 by the NRES Southampton REC A (11/SC/0274).

The BHPS ran from 1991 to 2008, repeatedly interviewing adult members of households. The Survey was designed to include more than 5,000 households, providing approximately 10,000 individual interviews. The UKHLS expanded the BHPS sample, with approximately 45,000 individuals, and from Wave 2 explicitly incorporated BHPS members. This analysis employs the first 7 waves of UKHLS data, spanning 2009 to 2017. Together the two surveys provide the potential to cover 26 years of health development. Our analysis uses the Great Britain (England, Scotland and Wales) general population samples for the BHPS and UKHLS (and includes the post-1999 Scotland and Wales boost samples). We restricted the sample to Great Britain to facilitate the maximum length of follow-up. Including the Northern Irish component would have required us to only use 15 years of data. More detail on the survey timelines and measures can be found in the user guides [24–26].

### Mental ill-health

The outcome considered in this study is the General Health Questionnaire (GHQ), which is a commonly used measure of psychological distress, covering domains of common mental disorders such as depression and anxiety [27–29]. This analysis employs the ‘short’ 12-item GHQ which involves asking respondents a series of questions relating to how they have been feeling over the last few weeks. The 12 items cover positive aspects such as ‘Have you recently been able to enjoy your normal day-to-day activities?’ which are scored on a scale of 1 ‘more so than usual’ to 4 ‘much less than usual’, as well as negative aspects such as ‘Have you recently been losing confidence in yourself’ which are scored on a scale from 1 ‘not at all’ to 4 ‘much more than usual’. The GHQ scale employed for this analysis is computed by re-scoring the items from 1–4, to 0–3 before summing to create an index from 0–36, where higher scores are indicative of more distress, and a worse mental state.

Although the validity of the GHQ as a screening instrument for psychiatric morbidity has been challenged [30], we do not operationalise the GHQ-12 on a case basis, rather as a continuum of mental distress for the purpose of within and between individual comparisons. Additionally, the presence of the item at every wave of the BHPS and UKHLS with consistent phrasing and scoring provides a valuable resource for exploring trajectories of mental ill-health over a long period.

### Age and cohort

We aim to evaluate trajectories in mental ill-health to provide a baseline knowledge of age and cohort trends over time, and in doing so demonstrate the utility of multilevel models as a non-parametric technique for such analyses. Our multilevel approach detailed below includes ages and cohorts as random effects, where the model treats them as category identifiers. The age range of the sample is restricted to those aged between 18 and 90, covering the majority of adulthood and ensuring a large sample size at all age-points. Each individual year is treated as a unit of the age random effect level, creating 73 age-units in total. Cohorts are measured by the respondent’s birth-year which forms the units of the cohort random level. We restrict the samples to cohorts with at least 150 observations to improve the analysis and reduce stochastic variation.

## Methods

Multilevel models are utilised to explore age and cohort effects in mental ill-health over time. This analysis seeks to assess the baseline variability of this health measure, using null models without controlling for any covariates which may explain the identified temporal patterns. Models are run for the full sample and additionally in separate analyses for females and males to identify any main differences in patterning by sex.

We treat ages and cohorts as random classifications within which individuals are nested. This technique effectively assesses the *general contextual effect* [31] of ages and cohorts as temporal contexts, in the same way that you would evaluate a spatial context such as a neighbourhood using multilevel modelling. In this way, we can assess temporal patterns in age and by cohorts through the predicted random effects without having imposed a parametric shape on the time variables, as would be the case if they were included as fixed effects.

**Fig 1:**
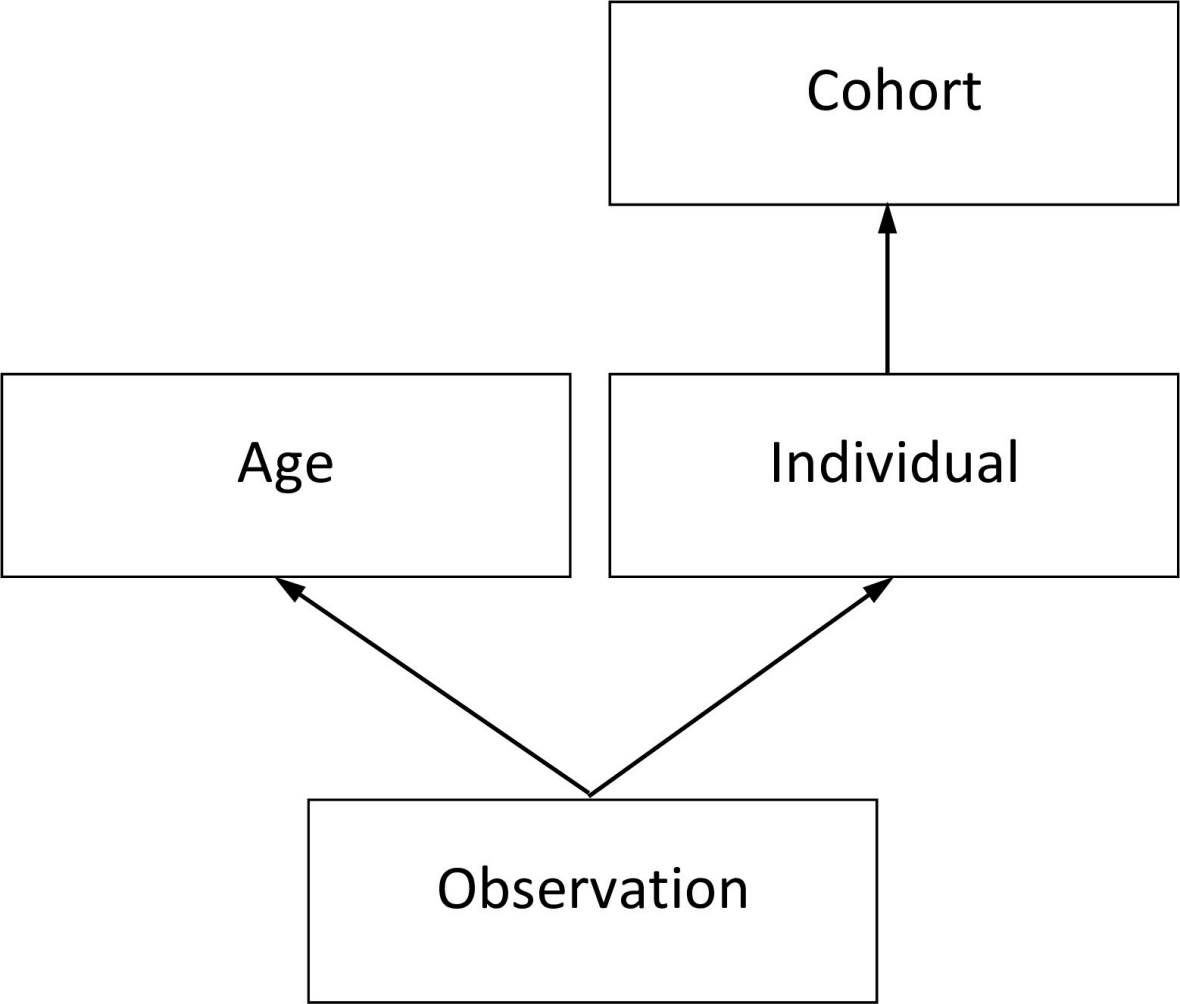
Multilevel data structure.

We independently test age and cohort random effects in separate models, before jointly including them in a single model to assess their trajectories, each accounting for the influence of the other. Note that each cohort has a varying age range as the panel is unbalanced and data collection for all participants does not start at the same age. Additionally, the separate sex samples have later starting dates for their cohort ranges (1909 for females, 1912 for males) than the full sample which begins at 1907. This is because, taken separately, there are not enough males or females born in the earliest years to meet our threshold of 150 observations. The cross-classified data-structure is detailed in Fig 1 and the number of units at each level is shown in Table 1.

**Table 1:** Data structure information for each sample and outcome.

*Sample*		Full	Females	Males
*Number of units at each level*	*Cohort (l)*	91	89	85
	*Age (k)*	73	73	73
	*Individuals (j)*	69,071	37,022	31,914
	*Observations (i)*	406,238	223,366	182,171
*Cohort range*		1907–1997	1909–1997	1912–1997
*Age range*		18–90	18–90	18–90
*Mean observations per person per cohort*		1.4–13.4	1.4–14.5	1.3–13.1

Eqs 1 and 2 detail the most complex model, with the intercept term (*β*_0_), all random effects (*μ*) included, and the lowest level random term (*e*_*i*_) signifying the observation residuals. The individual random level accounts for dependency in observations from the same respondent over time. The subscripts *i*, *j*, *k*, and *l* indicate the observation, individual, age, and cohort levels respectively.

$$GHQ_{ijkl}=\beta_{0ijkl}+\mu_{l}+\mu_{k}+\mu_{j}+e_{i}$$ (1)

$$\mu_{l}\sim N\left(0,\sigma^{2}{}_{u\left(l\right)}\right),\;\mu_{k}\sim N\left(0,\;\sigma^{2}{}_{u\left(k\right)}\right),$$

$$\mu_{j}\sim N\left(0,\;\sigma^{2}{}_{u\left(j\right)}\right),\;e_{i}\sim N\left(0,\;\sigma^{2}{}_{e}\right)$$ (2)

All models use Markov Chain Monte Carlo (MCMC) methods in MLwiN version 3.01 [32, 33], with runs of 50,000 iterations and a burn-in period of 2,000. This was sufficient to achieve convergence and to ensure a reasonable estimated sample size of over 200 for all parameters. To improve model run-time and convergence, orthogonal parameterisation and hierarchical centring, centred on the level with the fewest categories [32], was used. Models were sequentially fitted starting from a two-level model of observations nested within individuals, and the final models were verified through comparison of the Deviance Information Criterion (DIC), a measure of badness of fit penalised for model complexity, which is suitable for use in the comparison of MCMC output.

## Results

**Fig 2:**
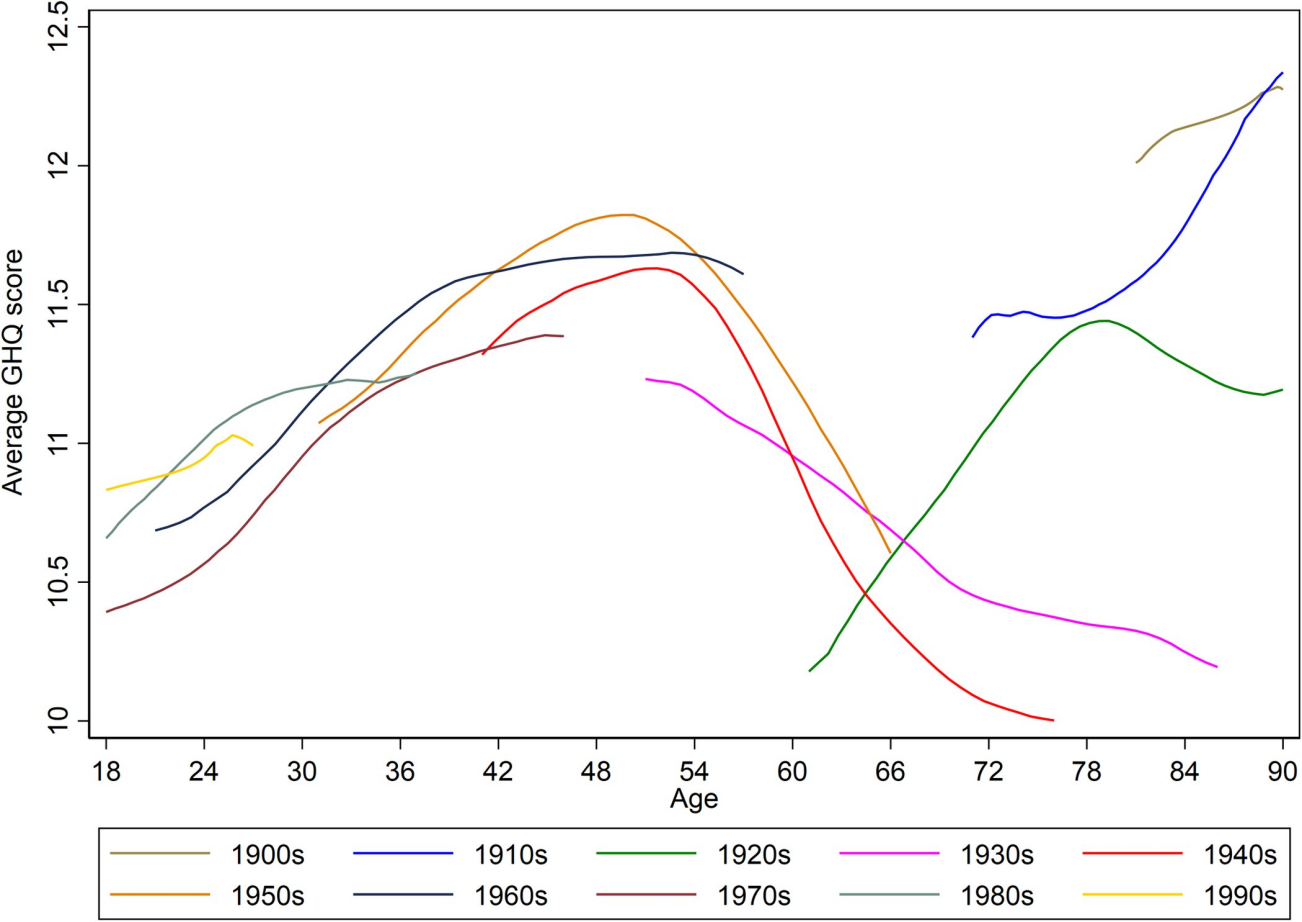
Average mental ill-health score by age, summarised by cohort decade. Higher scores on the GHQ measure are indicative of a worse mental state.

As an initial exploration of the age and cohort patterning of the data, we plot descriptive summaries of the general trends in mental ill-health score, averaged over decades of cohort-years. These are presented in Fig 2, where higher scores represent worse mental distress. Looking at the overall shape of the trend lines we can see that mental ill-health shows deteriorating scores towards middle-age, followed by improvements till around age 65 or 70 then worsening again. Also shown is how some decades–such as the 1970s –appear to trend with lower mental distress than others with its line sitting noticeably lower than that for later cohorts (1980s and 1990s) between the ages of 18 and 30.

Results for the model containing both age and cohort random classifications for the full and separate sex samples are presented in Table 2. The table also includes the Variance Partitioning Coefficient (VPC), which reports the proportion of variation attributed to each random level. It is calculated as the variance at any given level, divided by the total variance across all levels [34]. A model containing an interaction classification (age*cohort)–assessing if there were different age effects for different cohorts–was additionally tested (S1 Table). This model offered a slight improvement to model fit based on the DIC over the final model presented in Table 2, however, the variance explained was very small (0.1%) and any interaction patterning was not prominent (S1 Fig). Therefore, we do not present model results with the interaction classification but note the capacity of the multilevel approach to incorporate more complex dimensions.

**Table 2:** Model results including the median estimated coefficient and 95% credible intervals.

*Response*: *Mental ill-health*		*Full Sample*			*Females*			*Males*		
			*Credible Interval*			*Credible Interval*			*Credible Interval*	
		*β*	*2*.*5%*	*97*.*5%*	*β*	*2*.*5%*	*97*.*5%*	*β*	*2*.*5%*	*97*.*5%*
*Fixed Part*	Intercept	11.259	11.132	11.382	11.744	11.629	11.858	10.605	10.478	10.734
*Random Part*	Cohort	0.067	0.037	0.108	0.071	0.034	0.123	0.026	0.004	0.056
	Age	0.208	0.145	0.299	0.142	0.097	0.206	0.252	0.173	0.363
	Individual	13.902	13.706	14.101	14.585	14.29	14.866	12.357	12.098	12.619
	Observation	16.335	16.256	16.415	18.22	18.105	18.337	14.021	13.921	14.12
*Variance Partitioning Coefficient*	Cohort	0.2%			0.2%			0.1%		
	Age	0.7%			0.4%			0.9%		
	Individual	45.6%			44.2%			46.4%		
	Observation	53.5%			55.2%			52.6%		

**Fig 3:**
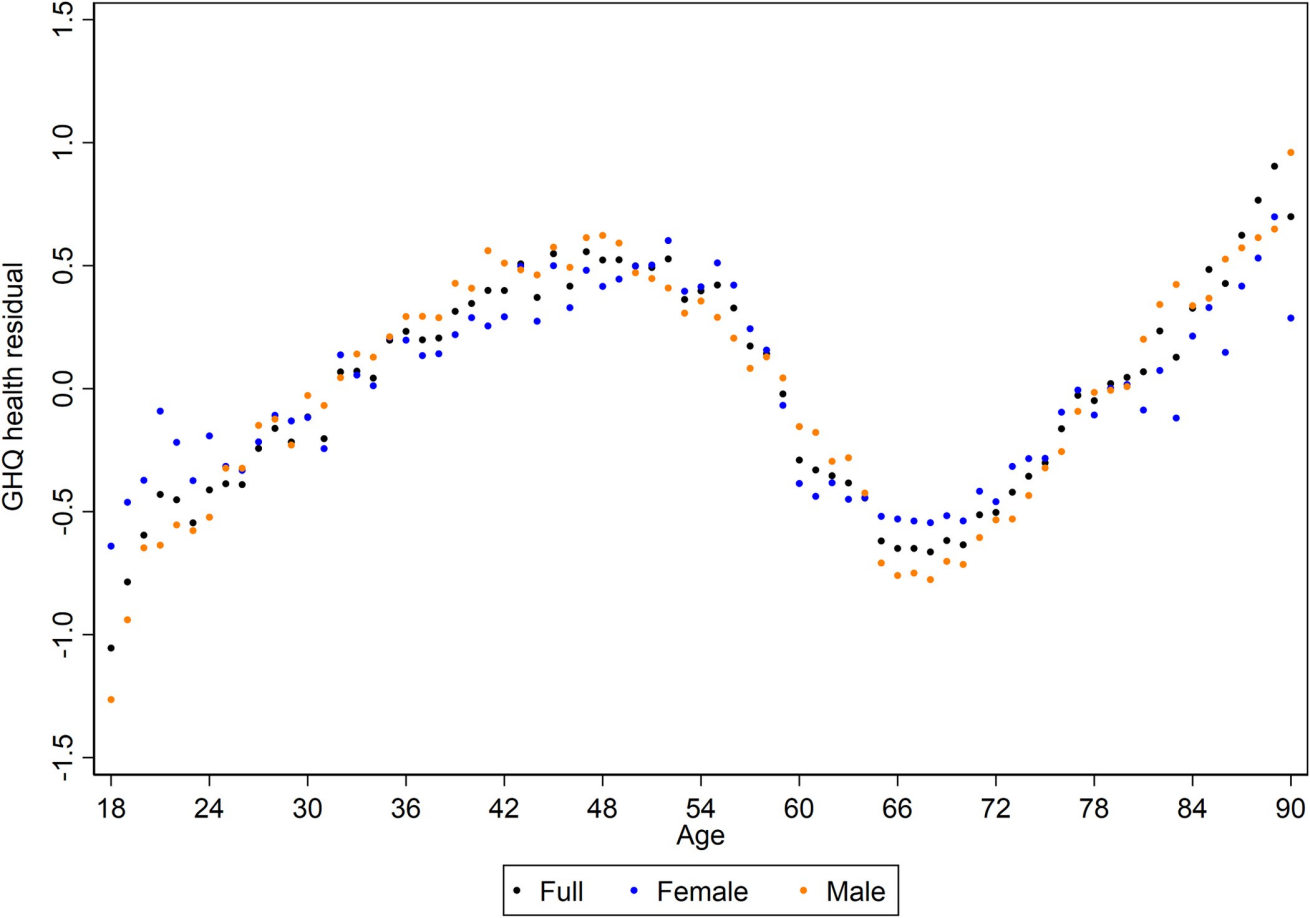
Estimated age random residuals for mental ill-health for each sample. Higher scores on the GHQ measure are indicative of a worse mental state.

**Fig 4:**
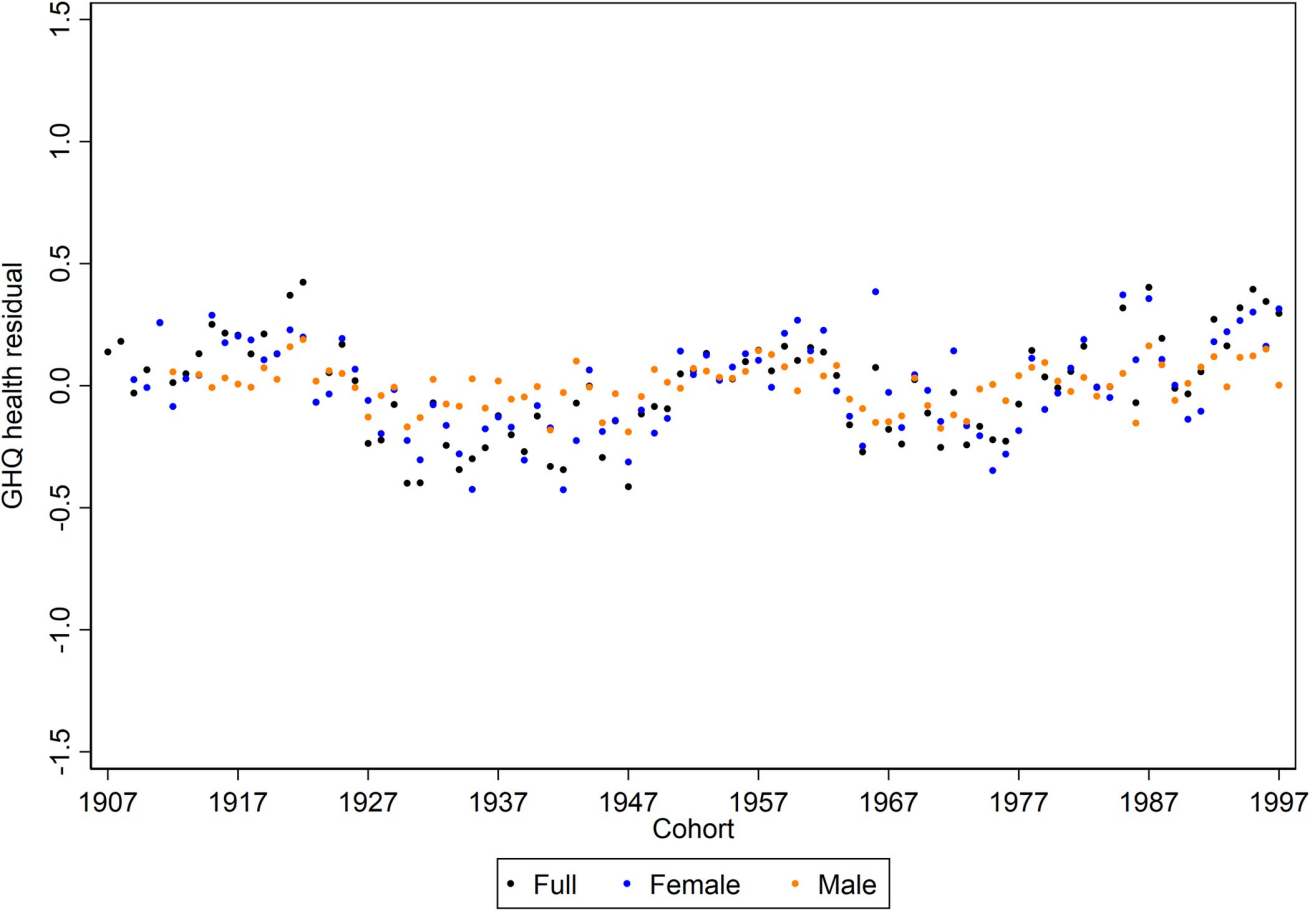
Estimated cohort random residuals for mental ill-health for each sample. Higher scores on the GHQ measure are indicative of a worse mental state.

Taking the full sample results as an example, Table 2 demonstrates that there is a high degree of intra-individual variability in observations of mental ill-health (53.5%), as well as a considerable degree of inter-individual variation (45.6%). Thus, there are some individuals who tend to suffer worse mental ill-health than others (accounting for between individual variation), but individuals are not necessarily trapped in one particular state and will experience periods of relatively worse or better mental health (variation within persons over time). Very little of the total variation in GHQ scores is attributed to the temporal contexts of ages and cohorts (0.7% and 0.2% respectively). Although the difference in variance explained is small, it is the case that the process of ageing is more relevant than shifts in cohort. This can clearly be seen in Figs 3 and 4 which show the estimated age and cohort random residuals from the final models. There is a greater range of values present across the age values than there is present for cohorts, the residuals of which are bounded within an expected 1-point shift on the GHQ scale.

The figures indicate a remarkable degree of patterning given that we are using a modelling approach that does not impose any structure on the data. Whether assessed on the full sample or separately for females or males, the estimated random residuals for age display a cubic pattern, whereby mental distress increases through to middle-age where there is an improvement to around the age of 65, where mental state appears to worsen again. This patterning reflects Fig 2. It should be noted these differences across the lifecourse are relatively small in terms of expected change in GHQ score, the total range is within 2-points. This alongside the small amount of variation accounted for by age (at most 0.9% in the males only sample) points again towards age as a temporal context being less important than other within and between individual effects.

Finally, in the estimated random residuals for cohort effects, the patterning is less pronounced and with smaller expected variation. For the full and female only samples, there appears to be improvements to mental health for some cohorts, notably the cohort-years of the 1930s, 1940s and 1970s. In contrast, for more recently born cohorts the mental distress trend appears to be worsening, which supports Fig 2. The differences remain marginal, however, and for the males only sample, there is a lack of patterning, which is reflected in only 0.1% of variation in GHQ score attributed to the cohort level.

## Discussion

The results of this exploratory analysis reveal a powerful technique for the detection of inherent temporal patterning in survey data. Our results for mental ill-health stand in opposition to the discourse of a u-shape in mental well-being and illness over the lifecourse. Instead, when the ageing trend is non-parametrically assessed, taking account of the influence of cohort differences, a cubic-shaped pattern is exposed. Mental ill-health worsens over time from young adulthood to around the age of 50, improving till around retirement age (~65) where it appears to decline through old age (see Fig 3). The overall trajectory is similar to that observed by Bell [35] who also used the BHPS dataset and controlled for cohort trends; it is reassuring to replicate this pattern by age even when including the additional UKHLS sample.

The suggestion of heightened mental distress at older ages supports previous research indicating depressive symptoms increase with age [13]. However, it should be recognised that as we use the GHQ, a self-reported indicator of mental distress, an increased manifestation of physical symptoms of mental illness and depressive symptoms at older ages [36] may be influencing the trends through later life for participants. This may particularly be the case as these exploratory models are unadjusted for covariates such as physical health status.

A benefit of the multilevel approach taken here is the capacity to gain a direct appraisal of the variation attributed to age and cohort as temporal contexts. The vast majority of variation in mental ill-health was positioned between individuals (46.5% in the full sample) and between observations within individuals (53.5% in the full sample). The strength of these inter- and intra-individual effects is a typical finding from longitudinal studies of mental illness and wellbeing, and has previously been observed for data from the BHPS [35, 37]. Studies from different contexts also show the strength of these effects. For example, VPC estimates of approximately 47% of variation position within individuals, and 51% between individuals are observed for a longitudinal model of adolescent mental health in Korea [38]. Future research could utilise the technique showcased here to explore which other factors, beyond age and cohort, may have a more potent impact on explaining variation in mental ill-health.

Our results highlight that–despite the importance usually attached to ageing processes–age accounted for very little of overall variation in mental distress (0.7% in the full sample). A higher proportion of variance at the age-level would have indicated that the process of growing older had a potent impact in the on-going development of mental ill-health. However, the low VPC observed for age (combined with the high inter- and intra-individual variation) instead suggest that trends in mental ill-health are highly variable and individualised. Therefore, whilst the multilevel approach employed reveals a distinct ageing trend in Fig 3, observations at a given age will show a spread of values in response to a multitude of other social, behavioural and environmental factors acting within and between individuals.

Additionally, it is known that mental health is a labile phenomenon and responsive to recent events and experiences [22], as well as being impacted by changing personal circumstances over the lifecourse, for instance through shifting employment stresses, or changing family dynamics. Given this and that the available measure of mental ill-health–the GHQ–asks participants to consider how they have been feeling over the last few weeks, this analysis is likely picking up changes in life circumstances and experiences which impact on a dynamic mental response, as evidenced in the high degree of between- and within-individual variation. It may, therefore, have been harder to pick up on age or cohort trajectories in a longer-term underlying mental state.

There was little evidence for distinct cohort trending in mental ill-health; the estimated range in random residuals was small, and cohort as a temporal context explained a small amount of the total variation in GHQ score. Cohort effects, at least in the context of the Great Britain sample analysed, appear not to have a strong influence on mental ill-health. If we had found a higher VPC for cohort this would have given weight to the importance of wider societal and cultural changes in mental illness trends; those born in the same year would be following more similar trends given shared cohort experiences. In contrast, because we report a very low VPC for cohort this means that those born contemporaneously are presenting differing GHQ scores, and may display divergent trajectories.

However, evaluation of the combined age and cohort influences hints at trending groups of cohort-years as either having relatively better or worse mental health over time, exposing those born in the 1930s, 1940s and 1970s as tracking over time with lower levels of mental distress. Additionally, the youngest cohort years tend towards higher levels of mental distress, though the differentiation from older cohorts is limited. This finding is in-keeping with studies which have emphasised the current crisis in the mental health of young persons [18]. Our results are helpful in presenting preliminary evidence for the role of cohorts in the mental health of young adults, rather than the alternative explanation that this age group has always suffered worse mental distress over time. Individuals born in the 90s cohorts would have been entering adulthood and the job market during a period of recession and austerity post 2008 financial crash, this socioeconomic context could be a possible explanation for the cohort patterning in mental ill-health [39]. However, given the limited power of cohorts in explaining total variation and the narrow differentiation of this group, this result should not be given too much weight at present.

The limited power of cohorts suggests that these effects may not be critical to the study of mental ill-health trajectories; the potential confounding may be small. However, it is vital to recognise that this implication relates to the GHQ operationalisation of mental ill-health. This is a combined measure using a range of information from both positive and negative aspects of wellbeing and covering features of common mental disorders. Future research could help determine whether cohort influences are more prominent for measures representing more specific conditions such as depression. Lifecourse research has indicated that early life and developmental contexts can play a role in pathways to adult depression [40]. Additionally, results from surveys in countries undergoing more rapid social and economic change could also demonstrate stronger cohort influences on mental illness. Further work implementing the technique showcased here would help reveal these potentialities.

This exploratory analysis focuses on ageing and cohort effects. However, it is possible that what we identify could in part reflect period effects, that is events at a certain point-in-time that influence all persons, regardless of their age or cohort. For example, the implementation of a distinct change in welfare policy. However, we anticipate that in examining trajectories of mental ill-health, continuous, trending period influences across time affecting all ages are unlikely [35]. For instance, whilst we can postulate that mental health may suffer a decline alongside an economic recession, the impacts of this are not likely to be even across all ages simultaneously, with life position at the time of such economic turmoil, as determined by cohort membership, offering a more plausible explanation for trends in health.

The strength of this analysis is in exposing the power of multilevel modelling to reveal underlying temporal trends. Demonstrated with application to a major heath dimension, the technique allows the data to speak for itself without *a priori* imposing a parametric structure on expected trajectories. The results highlight the remarkable patterning present in ageing trajectories in particular. However, we also observed that these temporal effects were relatively unimportant compared to other between and within effects in explaining variations in mental ill-health. Health researchers would benefit from exploiting this methodology to explore a range of health outcomes over time and contribute to broader understandings of health trajectories.

## Supporting information

S1 Table Model results including the median estimated coefficient and 95% credible intervals for a model containing an age*cohort random classification. (DOCX)

S1 Fig Predicted mental ill-health (GHQ) score by age by cohort-year for the full sample for a model containing an age*cohort random classification. First row cohort-years highlighted in black left to right: 1990–1997; 1980–1989; 1970–1979. Second row cohort-years highlighted in black left to right: 1960–1969; 1950–1959; 1940–1949. Bottom row cohort-years highlighted in black left to right: 1930–1939; 1920–1929; 1907–1919.
(DOCX)
