# Supplementary material for: Ageing and cohort trajectories in mental ill-health: An exploration using multilevel models
Source: PLoS One. 2020 Jul 9;15(7):e0235594. doi: 10.1371/journal.pone.0235594 (PMC7347124; doi:10.1371/journal.pone.0235594)
Supplement: S1 Table — (DOCX) [file pone.0235594.s001.docx]

**S1 Table. Model results including the median estimated coefficient and 95% credible intervals for a model containing an age*cohort random classification.**

| *Response: Mental ill-health* | | *Full Sample* | | | *Females* | | | *Males* | | |
| --- | --- | --- | --- | --- | --- | --- | --- | --- | --- | --- |
|  |  |  | *Credible Interval* | |  | *Credible Interval* | |  | *Credible Interval* | |
|  |  | *β* | *2.5%* | *97.5%* | *β* | *2.5%* | *97.5%* | *β* | *2.5%* | *97.5%* |
| *Fixed Part* | Intercept | 11.256 | 11.135 | 11.379 | 11.74 | 11.622 | 11.857 | 10.603 | 10.477 | 10.733 |
| *Random Part* | Age*Cohort | 0.020 | 0.014 | 0.027 | 0.023 | 0.011 | 0.036 | 0.019 | 0.009 | 0.031 |
|  | Cohort | 0.060 | 0.033 | 0.100 | 0.066 | 0.03 | 0.115 | 0.024 | 0.004 | 0.055 |
|  | Age | 0.206 | 0.146 | 0.292 | 0.141 | 0.094 | 0.204 | 0.249 | 0.169 | 0.355 |
|  | Individual | 13.903 | 13.711 | 14.104 | 14.587 | 14.294 | 14.877 | 12.355 | 12.089 | 12.619 |
|  | Observation | 16.316 | 16.235 | 16.392 | 18.198 | 18.085 | 18.314 | 14.003 | 13.906 | 14.103 |
| *Variance Partitioning Coefficient* | Age*Cohort | 0.1% |  |  | 0.1% |  |  | 0.1% |  |  |
|  | Cohort | 0.2% |  |  | 0.2% |  |  | 0.1% |  |  |
|  | Age | 0.7% |  |  | 0.4% |  |  | 0.9% |  |  |
|  | Individual | 45.6% |  |  | 44.2% |  |  | 46.4% |  |  |
|  | Observation | 53.5% |  |  | 55.1% |  |  | 52.5% |  |  |
