# Supplementary material for: Ageing and cohort trajectories in mental ill-health: An exploration using multilevel models
Source: PLoS One. 2020 Jul 9;15(7):e0235594. doi: 10.1371/journal.pone.0235594 (PMC7347124; doi:10.1371/journal.pone.0235594)
Supplement: S1 Fig — First row cohort-years highlighted in black left to right: 1990–1997; 1980–1989; 1970–1979. Second row cohort-years highlighted in black left to right: 1960–1969; 1950–1959; 1940–1949. Bottom row cohort-years highlighted in black left to right: 1930–1939; 1920–1929; 1907–1919. (DOCX) [file pone.0235594.s002.docx]

**S1 Fig. Predicted mental ill-health (GHQ) score by age by cohort-year for the full sample for a model containing an age*cohort random classification.** First row cohort-years highlighted in black left to right: 1990-1997; 1980-1989; 1970-1979. Second row cohort-years highlighted in black left to right: 1960-1969; 1950-1959; 1940-1949. Bottom row cohort-years highlighted in black left to right: 1930-1939; 1920-1929; 1907-1919.

**
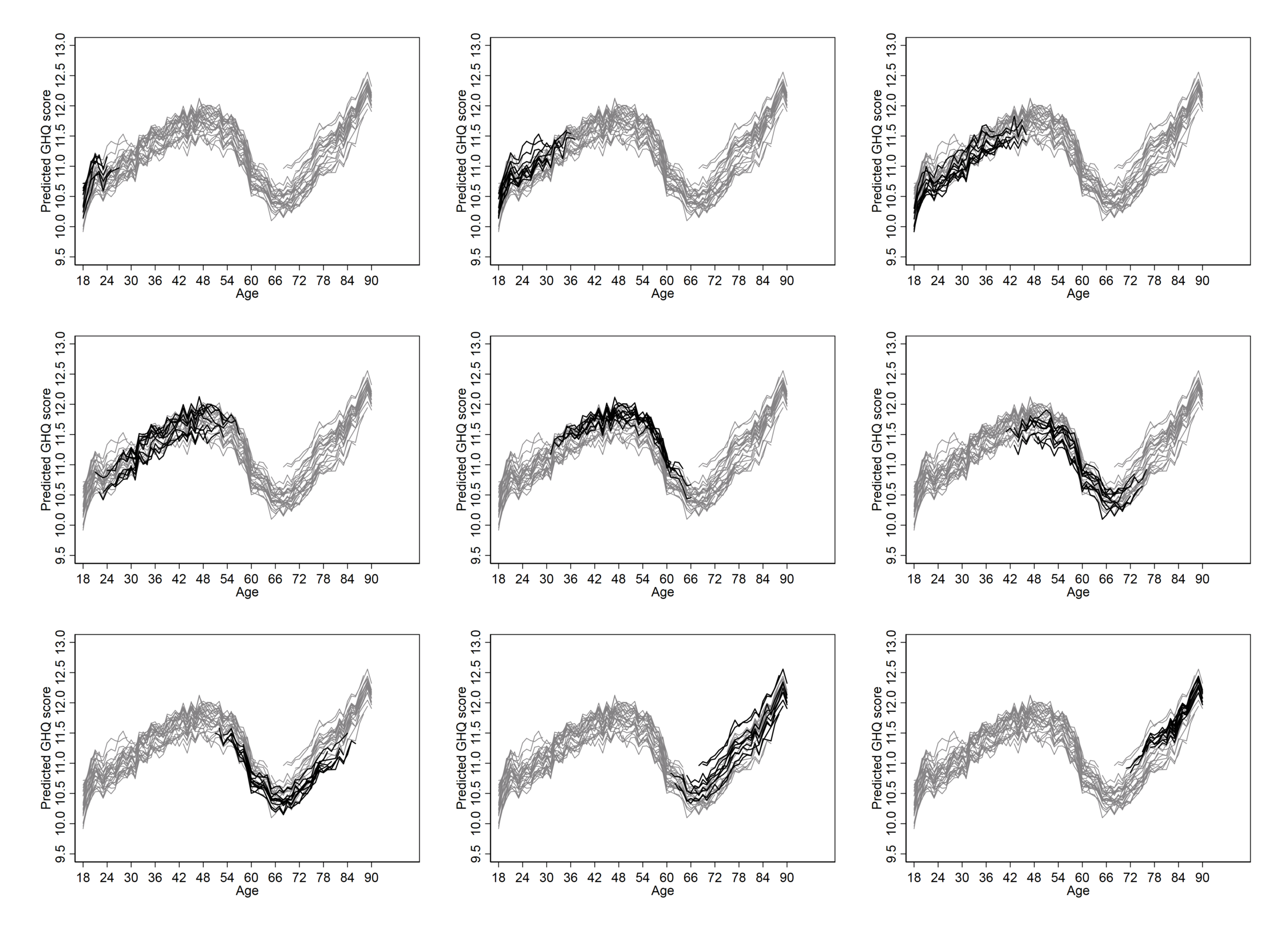
**
